# Needs-led human resource planning for Sierra Leone in support of oral health

**DOI:** 10.1186/s12960-021-00623-x

**Published:** 2021-09-01

**Authors:** Swapnil Gajendra Ghotane, Patric Don-Davis, David Kamara, Paul R. Harper, Stephen J. Challacombe, Jennifer E. Gallagher

**Affiliations:** 1 Faculty of Dentistry, Oral & Craniofacial Sciences At King’s College London, Centre for Host Microbiome Interactions, Denmark Hill Campus, Bessemer Road, London, SE5 9RS United Kingdom; 2 College of Medicine and Allied Health Sciences, Connaught Hospital, Freetown, Sierra Leone; 3 Oral Health Department, Connaught Hospital, Freetown, Sierra Leone; 4 School of Mathematics, Cardiff University, Cardiff, CF24 4AG UK; 5 Faculty of Dentistry, Oral and Craniofacial Sciences At King’s College London, Centre for Host Microbiome Interactions, Guys Campus, London, SE1 9RT UK

**Keywords:** Oral health, Oral health workforce, Dental workforce, Operational research, Oral health needs, Treatment needs, Low-income countries, Dental therapists, Atraumatic restorative treatment, Tooth extraction, ICCMS, ICDAS

## Abstract

**Background:**

In Sierra Leone (SL), a low-income country in West Africa, dental care is very limited, largely private, and with services focused in the capital Freetown. There is no formal dental education. Ten dentists supported by a similar number of dental care professionals (DCPs) serve a population of over 7.5 million people. The objective of this research was to estimate needs-led requirements for dental care and human resources for oral health to inform capacity building, based on a national survey of oral health in SL.

**Methods:**

A dedicated operational research (OR) decision tool was constructed in Microsoft Excel to support this project. First, total treatment needs were estimated from our national epidemiological survey data for three key ages (6, 12 and 15 years), collected using the ‘International Caries Classification and Management System (ICCMS)’ tool. Second, oral health needs were extrapolated to whole population levels for each year-group, based on census demographic data. Third, full time equivalent (FTE) workforce capacity needs were estimated for mid-level providers in the form of Dental Therapists (DTs) and non-dental personnel based on current oral disease management approaches and clinical timings for treatment procedures. Fourth, informed by an expert panel, three oral disease management scenarios were explored for the national population: (1) *Conventional care (CC)*: comprising oral health promotion (including prevention), restorations and tooth extraction; (2) *Surgical and Preventive care (S*_*5&6*_*P and S*_*6*_*P)*: comprising oral health promotion (inc. prevention) and tooth extraction (D5 and D6 together, & at D6 level only); and (3) *Prevention only (P)*: consisting of oral health promotion (inc. prevention). Fifth, the findings were extrapolated to the whole population based on demography, assuming similar levels of treatment need.

**Results:**

To meet the needs of a single year-group of childrens’ needs, an average of 163 DTs (range: 133–188) would be required to deliver *Conventional care (CC)*; 39 DTs (range: 30–45) to deliver basic *Surgical and Preventive care (S*_*6*_*P)*; 54 DTs for more extended *Surgical and Preventive care (S*_*5&6*_*P)* (range 38–68); and 27 DTs (range: 25–32) to deliver *Prevention only (P)*. When scaled up to the total population, an estimated 6,147 DTs (range: 5,565–6,870) would be required to deliver *Conventional* care *(CC)*; 1,413 DTs (range: 1255–1438 DTs) to deliver basic *Surgical and Preventive care (S*_*6*_*P)*; 2,000 DTs (range 1590–2236) for more extended *Surgical and Preventive care (S*_*5&6*_*P)* (range 1590–2236); and 1,028 DTs to deliver *Prevention* only *(P)* (range: 1016–1046). Furthermore, if oral health promotion activities, including individualised prevention, could be delivered by non-dental personnel, then the remaining surgical care could be delivered by 385 DTs (range: 251–488) for the *S*_*6*_*P* scenario which was deemed as the minimum basic baseline service involving extracting all teeth with extensive caries into dentine. More realistically, 972 DTs (range: 586–1179) would be needed for the *S*_*5&6*_*P* scenario in which all teeth with distinctive and extensive caries into dentine are extracted.

**Conclusion:**

The study demonstrates the huge dental workforce needs required to deliver even minimal oral health care to the Sierra Leone population. The gap between the current workforce and the oral health needs of the population is stark and requires urgent action. The study also demonstrates the potential for contemporary epidemiological tools to predict dental treatment needs and inform workforce capacity building in a low-income country, exploring a range of solutions involving mid-level providers and non-dental personnel.

## Background

### Sierra Leone (SL) and King’s Centre for Global Health (KCGH)

Sierra Leone (SL) has a population of over 7.5 million people, 41% of whom are aged under 15 years [1]. This West African country has been recovering from the fallout of a decade long civil war (1991–2002), Ebola virus disease (EVD) (2014–2016) and natural disasters (2017), resulting in considerable loss of human life which further weakened their health system [2–4]. The country is administratively organized into four regions and 16 districts. King’s Centre for Global Health (KCGH) has active health partnerships internationally in low-income countries (LICs), including SL, working with government and local agencies in a research informed manner and supporting in the process of re-building health care delivery in a sustainable manner [5], including capacity building initiatives.

### Oral health and disease

Oral diseases, notably dental caries and periodontal disease are among the major contributors to the global burden of chronic diseases [6]; their prevalence is increasing, especially in sub-Saharan Africa [7]. Dental caries affects 60–90% of children and almost all adults worldwide [8]. Previous localised epidemiological studies within SL suggested considerable levels of dental caries in children (60% to 80%) and adults (around 80%) [9–11].

Working in partnership, researchers from King’s College London and public sector dentists from SL conducted the first national oral health survey of schoolchildren aged 6, 12 and 15 years in 2017 [12]. Dental caries was the predominant oral disease: over 82% of the 1,174 schoolchildren surveyed had dental caries experience (including visual changes in enamel) [12]. In addition, out of the total schoolchildren surveyed, 10% reported current pain and 7–8% had oral conditions related to untreated caries, i.e. PUFA (Pulp, Ulceration, Fistula, Abscess) lesions [12]. Prior to embarking on the national survey, the views of dental professionals and key individuals associated with SL were sought on the challenges and solutions for Sierra Leone, together with their rationale for working in the country and vision for the future [13].

### Oral and dental workforce

SL has a dearth of dental professionals as only 10 dentists, supported by approximately 10 dental care professionals (dental therapists, dental assistants and oral health promoters) are available nationally; almost all of whom are based in the capital city Freetown [13]. A critical shortage of dental personnel has been evident since 1963 [14, 15], with only one dentist to 200,000 people at best over that time (Table 1).

**Table 1:** Dental workforce in Sierra Leone: 1963–2020

Era	Year	Dentists	Dental therapists (DTs)	Dental nurses/assistants/oral health promoters	Population(in million)	Dentist: Population ratio
Pre-civil war	1963	10	n/a	10	2.2	1: 220,000
	1984	18	n/a	n/a	3.4	1: 188,889
	1989	16	2	5	3.9	1: 243,750
Post-civil war	2017	14	6	6	7.1	1: 507,143
	2020	10	5	5	7.5	1: 750,000

See Refs. [14, 15, 44]

Thorpe S. Dental personnel registered with the Medical and Dental Council of Sierra Leone. 2018

n/a: not available

Our current evidence [13] suggests that some additional oral health care is delivered through personnel with no formal dental qualification i.e. non-dental personnel (NDPs) including community health officers (CHOs), community health workers (CHWs) and traditional healers as well as visiting non-governmental organizations.

**Fig. 1:**
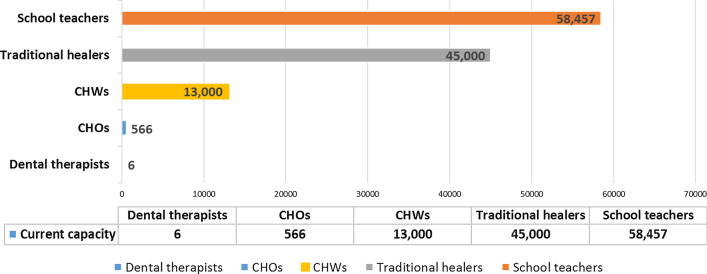
Current capacity of Dental Therapists and Non-dental personnel (NDP) including Community Health Officers (CHOs), Community Health Workers (CHWs), Traditional healers and Schoolteachers available in Sierra Leone. Sources: Thorpe S. Dental personnel registered with the Medical and Dental Council of Sierra Leone. 2018 (pers comm); World health Organisation [WHO]. Towards health systems resilience: Strategic investments for a needs-based health workforce in Sierra Leone. 2015. Ministry of Health and Sanitation (MoHS). Sierra Leone Basic package of Essential Health Services 2015–2020. [Available from: https://mohs2017.files.wordpress.com/2017/06/gosl_2015_basic-package-of-essential-health-services-2015-2020.pdf]. Dziewanki D. How traditional healers helped defeat Ebola. [Electronic]. 2015 [Available from: https://www.aljazeera.com/indepth/features/2015/10/traditional-healers-helped-defeat-ebola-151028114811599.html. Accessed on: 05 March 2020.]. United Nations Educational Scientific and Cultural Organization (UNESCO) Institute for Statistics. Education: National Monitoring: Classroom teachers and academic staff by sex: Number of teachers by teaching level of education. [Available from: http://data.uis.unesco.org/. Accessed on: 18 June 2019]

CHOs serve as primary health care providers working in an estimated 1,200 Peripheral Health Units (PHUs) operating under the Ministry of Health and Sanitation [16, 17]. Similarly, CHWs also provide basic care at primary care level although they have less training compared with CHOs [18]. There are an estimated 566 CHOs and more than 13,000 CHWs in SL as shown in Fig. 1 [18]; however, the extent of any oral health care within their scope of practice is undocumented and considered to be low.

In addition, traditional healers including herbalists and religious healers, advise and treat patients either part-time, typically from their own homes, or as a full-time job from stand-alone facilities [19]. An estimated 45,000 traditional healers are operating nationally [20], greatly exceeding the number of community health personnel.

### Operational research (OR) models for addressing human resources for oral health

For any health system, human resources for health (HRH) are crucial [21, 22], including oral and dental personnel [23]. However, inequalities in the availability and distribution of HRH exist between and within countries and tend to be more prominent in low-income countries (LICs) [24–27]. There has been an increasing trend to use operational research (OR) models in health services research in recent decades [28–30]. The World Health Organization (WHO) defines OR as “research that helps to identify solutions to problems that limit quality, efficiency and effectiveness, or to determine which alternative service delivery strategy would yield the best outcome” [31]. There are three fundamental stages in OR which constitute the foundation of OR modelling in any field as follows: problem identification, model development; and simulation [32]. OR facilitates representing real systems whilst allowing the effect of different scenarios to be explored within the model [33].

Workforce estimates generated through OR aid planning [34], and address combinatorial complexity usually associated with HRH [35]. Whilst OR has been used to estimate dental workforce in high-income countries [28, 30, 36], and some low–middle-income settings [37, 38], no study to the knowledge of the authors has used OR to estimate dental workforce needs in a low-income country, often because of a lack of reliable and relevant oral health data [28].

Multiple challenges in delivering oral health care were demonstrated by dentists working in Sierra Leone [13], together with the vision of developing mid-level providers in the form of dental therapists with an appropriate scope of practice [13, 39]. Thus, it is imperative to have baseline estimates of human resources for oral health, based on evidence of treatment needs [12], to inform capacity building. Therefore, this study intended to address the following research questions using data from the national oral health survey of children in SL:

1. What dental therapist (DT) capacity is required across different scenarios of oral disease management, to support the vision of meeting the oral health needs of Sierra Leoneans, through developing mid-level providers?
2. What non-dental personnel (NDP) capacity would be required to support DTs in delivering care?
3. What is the minimum baseline dental workforce capacity to manage current oral disease?

Hence, the aim of this study, was to inform the development of a robust oral health strategy including the development of a contemporary dental workforce and provision of oral healthcare.

## Methods

**Fig. 2:**
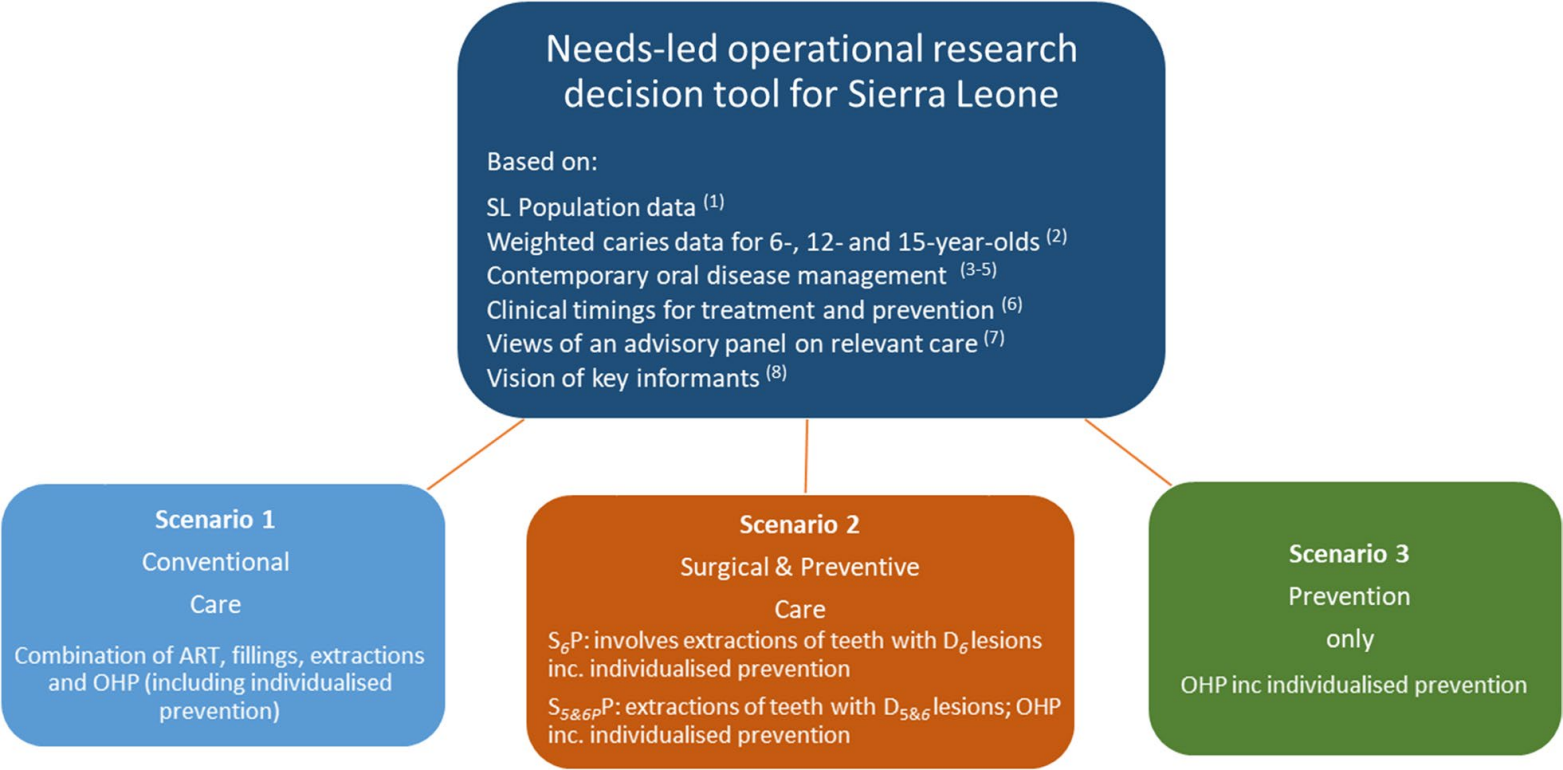
Oral Health Workforce Scenarios for Sierra Leone. Notes: ^1^ Statistics Sierra Leone. 2015 Population and Housing Census. [Available from: https://www.statistics.sl/images/StatisticsSL/Documents/Census/2015/sl_2015_phc_thematic_report_on_pop_structure_and_pop_distribution.pdf. Accessed on: 15 Feb 2017]. ^2^ Weighted data from a national level oral health survey of schoolchildren in SL conducted in 2017. ^3^ Ref. [40]. ^4^Ref. [42]. ^5^ Ref. [43]. ^6^ Ref. [41]: absolute timings of key dental procedures based on WTE-37.5 h for 46 weeks in a year. ^7^ Panel of members from the ICCMS, KCL and Public Sector Dentistry in SL. ^8^Ref. [13]

A project-specific operational research (OR) decision tool (Fig. 2) was constructed in Microsoft Excel to model dental caries in the SL population and to produce workforce estimates of dental therapists (DTs) and non-dental personnel (NDPs) against different scenarios of oral disease management.

The concept for this OR tool was informed by the ICCMS dental caries management system [40], clinical timings on treatment and prevention [41], current evidence on managing the oral disease using three scenarios [42, 43], and an advisory panel on relevant care for a low-income country which consisted of dental experts from King’s and the public sector in SL. The data for modelling were obtained from: (a) the SL national 2015 census [44]; (b) a weighted sample from a national oral health survey of schoolchildren [12, 45]; and, (c) the views of key individuals [13], as part of a programme of doctoral research [12].

First, estimates of treatment needs were established using recent dental caries data from three key age groups (6-, 12- and 15-year-old school children) in line with the ICCMS approach to disease management [40]. Second, total clinical hours required to deliver oral health promotion and basic preventive and surgical (tooth extraction) treatment by DTs were estimated, informed by past research relating to dentists and DCPs [41]. Third, the number of DTs needed to manage oral disease across the three key age groups nationally was calculated assuming that they would work full time (37.5 h a week) per year (46 weeks). Fourth, these findings were extrapolated to the national population [44], taking a similar approach to Wanyonyi et al. [30], to estimate the number of dental therapists (mid-level providers) required using three different scenarios outlined below to manage the needs ranging from basic conventional clinical care to prevention alone. Fifth, and finally, the number of NDPs needed to support oral health promotion working 1 day per week (7.5 h) over one year (46 weeks) was calculated, assuming similar clinical timings for oral health promotion and prevention as DTs. Further details are outlined below.

### Weighted proportion of dental caries data

Epidemiological data on the prevalence of dental caries were obtained through a national oral health survey of schoolchildren for three key age groups (6, 12 and 15 years) conducted in 2017 [45]. The caries diagnostic criteria for this survey were based on the International Caries Classification and Management System (ICCMS)’ (formerly termed as ICDAS) which reports dental caries at six levels from code 0 (visually no evidence of caries) to code 6 (extensive decay involving dentine) [40].

The data were weighted with respect to ‘age’ and ‘region’ across the four regions of SL in line with the 2015 census data [44]. Next, the average number of decayed teeth by ICCMS code (using the highest code for each tooth) was calculated for each of the three age groups of schoolchildren surveyed (Table 2). As the population of children in the 2015 census was reported in ‘five-year age bands’, one-fifth of the population of each ‘five-year age band’ was estimated to represent the total number of children for 6-(*n* = 221,743), 12-(*n* = 169,458) and 15-(*n* = 174,724) years, respectively, the variation in which may be explained by rising birth rates and childhood mortality.

**Table 2:** Prediction of treatment required at child and tooth level in 6-,12- and 15-year-olds in Sierra Leone based on dental caries management informed by ICCMS

Dentition	ICCMS^1^		Predicted treatment Procedure^1^	15-year olds				12-year olds				6-year olds			
	Decay description	Decay (D) code		% of children	Mean no. of decayed teeth	No. of children requiring treatment	No. of teeth requiring treatment	% of children	Mean no. of decayed teeth	No. of children requiring treatment	**No. of teeth** **requiring treatment**	**% of children**	**Mean no. of decayed teeth**	**No. of children** **requiring treatment**	**No. of teeth** **requiring treatment**
Primary	Extensive decay into dentine	D6	Tooth extraction									14%	2.02	31,044	62,709
	Distinct decay into dentine	D5										24%	1.99	53,218	105,904
	Underlying dentinalshadowing	D4	Atraumatic restorative treatment (ART)									50%	2.33	110,872	258,331
	Slight enamel breakdown	D3										66%	3.03	146,350	443,442
Permanent	Extensive decay into dentine	D6	Tooth extraction	17%	1.76	29,703	52,277	11%	1.31	18,640	24,419	1%	1.2	2,217	2,661
	Distinct decay into dentine	D5	Tooth filling	19%	1.46	33,198	48,468	14%	1.5	23,724	35,824	2%	1.0	4,435	4,435
	Underlying dentinal shadowing	D4	Tooth filling	50%	2.40	87,362	209,669	40%	2.45	67,783	166,069	13%	1.46	28,827	42,087
	Slight enamel breakdown	D3	ART	75%	3.85	131,043	504,516	63%	3.78	106,759	403,548	45%	1.80	99,784	179,612
	Visual change in enamel	D2	Prevention (Fluoride varnish)	54%	–	–	–	51%	–	–	–	28%	–	–	–
	No decay	D0	Prevention (Fluoride varnish)	6%	–	–	–	9%	–	–	–	36%	–	–	–
Total						174,724^4^	814,930^3^			169,458^4^	629,860^3^			221,743^4^	1,099,180^3^

^1, 2^Informed by an advisory panel on relevant care in LIC consisting of members from the ICCMS, KCL in the UK and COMAHS in SL; and Ref. [40]

^3^Total number of teeth requiring treatment with ICCMS decay codes from 3 to 6. Teeth with decay code 2 are excluded as treatment recommended is only preventative which is recommend for all children

^4^Total children in each of the three key age groups estimated from—Statistics Sierra Leone. 2015 Population and Housing Census. [Available from: https://www.statistics.sl/images/StatisticsSL/Documents/Census/2015/sl_2015_phc_thematic_report_on_pop_structure_and_pop_distribution.pdf. Accessed 15 Feb 2017]

### Prediction of treatment needs and treatment procedures

The predicted treatment procedures were informed by ICCMS [40], and an advisory panel on relevant care in a LIC consisting of King’s College London staff with clinical and epidemiological expertise in the UK and the SL public sector. It was estimated that teeth with decay code 6 (D6-‘extensive decay into dentine’) would require extraction as a treatment procedure. Similarly, teeth with code 5 (D5-distinct decay into dentine) and code 4 (D4-underlying dentinal shadowing seen in teeth) would require a tooth filling as the treatment procedure. Furthermore, it was estimated that teeth with decay code 3-(‘slight enamel breakdown’) could be treated using ‘atraumatic restorative treatment (ART) [46]. Finally, teeth with only ‘visual change in enamel’ (D2) and those with ‘no visual changes in enamel’ yet (D0 or D1) that could benefit from primary preventive measures such as ‘fluoride varnish’ [47], were estimated.

### Scenarios of oral disease management

The three management scenarios explored in this OR model are outlined below:

1. Conventional Care (CC): Based on the management of oral disease routinely seen in a clinical setting within primary care. The care elements in this scenario were proposed at three levels: ‘*Oral health promotion and prevention at child level’; ‘restoration at tooth level’* and *‘surgical (tooth extraction) treatment at tooth level’.*
  The estimated time to deliver all three elements of care was adapted through clinical timings for DTs reported in the UK study carried out in primary care [41]. ‘ Oral health promotion and individualised prevention at child level’ included measures such as oral examination, oral hygiene and diet advice, and fluoride varnish which were considered applicable and beneficial to all schoolchildren, as the proportion of children with no visual caries was very small across all three age groups. For oral health promotion (i.e. oral hygiene and diet advice), the estimated time required for advice was calculated assuming the procedure per school was delivered twice a year.
  Restoration at tooth level’ consisted of the estimated clinical hours required by DTs to deliver ‘ART’ for teeth with decay code 3 (D3) and ‘tooth fillings’ for teeth with decay codes 4 and 5 (D4 and D5).
  For ‘s*urgical (tooth extraction) treatment at tooth level’*, estimated clinical hours were calculated for carrying out primary and permanent teeth extractions. As extraction of permanent teeth is not normally within the scope of practice of DTs [48], the average time required by a dentist was utilised for estimating total clinical hours [41].
2. Surgical and Preventive (SP): This scenario focussed on surgical (teeth extraction) and preventative measures for oral disease management; however, no restoration component was considered. Two subtypes under this scenario were as follows:
  - S *6* P : In this approach, only teeth with decay code 6 (D6) were considered for extraction along with ‘Oral health promotion (OHP) and individualised prevention at child level’.
  - S *5*&*6* P: In this approach, teeth with decay codes 5 (D5) and 6 (D6) were considered for extraction along with ‘ Oral health promotion (OHP) and individualised prevention at child level’ .
3. Prevention (P) only: This scenario was solely based on preventative management of oral disease with no surgical or restorative procedures. The total clinical hours were estimated only from ‘*Oral health promotion (OHP) and individualised prevention at child level’* as detailed above.

## Results

### Treatment needs

Most children surveyed had evidence of disease and clinical treatment needs, together with a need for prevention. Estimated treatment needs for the population, by age, are presented in Table 2, with the model suggesting that over one million teeth requiring active treatment amongst the 6-year-old population. The level of need was lower in 12 year-old (629,860 teeth) as a result of having made the transition to their permanent dentition, rising by 29% in the 15-year-old population (814,930 teeth).

The most urgent treatment needs related to extraction of teeth, both primary and permanent. Seventeen per cent of 15-year-olds (*n* = 29,703) and 11% of 12 year-olds (*n* = 18,640) were projected to require at least one tooth extraction at D6 level, involving 52,277 and 24,419 permanent teeth, respectively.

Amongst 6-year-old children, the level of need was greatest in their primary dentition, with 38% children (*n* = 84,262) estimated to have heavily diseased primary teeth at D5 or D6 level collectively (*n* = 168,613 teeth), 62,709 of which were at D6 level only. Furthermore, in their permanent dentition up to 3% of 6 year-olds were estimated to require extraction of permanent teeth (*n* = 7,096) if all significantly affected teeth are extracted (D5 & D6) and 2,217 permanent teeth at D6 level alone.

In relation to tooth restorations, 69% of 15 year-olds and 54% of 12 year-olds were estimated to require one or more ‘tooth fillings’ for D4 and D5 lesions which corresponded to 258,137 and 201,893 teeth, respectively. Fifteen percent of 6 year-olds were estimated to need a tooth filling in approximately 46,522 permanent teeth.

Similarly, almost three-quarters of 15 year-olds (75%) were estimated to require one or more ‘ART’ restorations in some 504,516 teeth and 63% of 12 year-olds in 403,548 teeth. In 6 year-olds, some 701,773 primary teeth and 179,612 permanent teeth were estimated to require ‘ART’.

### Conventional scenario (CC)

In this scenario, to address the needs outlined in Table 2, a total of 291,157 clinical hours would be needed to treat the 15-year-olds population, 228,977 clinical hours for 12 year-olds and 323,663 clinical hours for 6 year-olds-old to enable DTs to treat current oral disease, as shown in Table 3. This translated into workforce requirement of 169 DTs to serve the 15-year-old population compared with 133 for 12 year-olds and 188 for 6 year-olds to manage disease conventionally including oral health promotion and prevention, restoration and extraction, as shown in Table 4. This represented an average of 163 DTs per year-olds-group.

**Table 3:** Prediction of clinical hours required to provide care at child and tooth level in 6-,12- and 15-year-olds in SL using dental therapists

Care type^1^	Treatment procedure^2^	Average time (mins) per procedure/tooth for DTs^3^	15-year olds (*n* = 174,724)			12-year olds (*n* = 169,458)			6-year olds^5^ (*n* = 221,743)		
			No. of children requiring treatment ^4^	No. of teeth requiring treatment ^4^	Estimated time (hours) for DTs	No. of children requiring treatment ^4^	No. of teeth requiring treatment^4^	Estimated time (hours) for DTs	No. of children requiring treatment ^4^	No. of teeth requiring treatment^4^	Estimated time (hours) for DTs
Oral health promotion and individualised prevention at child level	Oral examination	8	All children	All teeth	23,297	All children	All teeth	22,594	All children	All teeth	29,566
	Fluoride varnish (twice a year)^**7**^	3	All children	All teeth	17,472	All children	All teeth	16,946	All children	All teeth	22,174
	Oral hygiene advice	6.8	All children	7,671^**6**^ (schools)	1,739	All children	7,671^**6**^ (schools)	1,739	All children	7,671^**6**^ (schools)	1,739
	Diet advice	5.6	All children	7,671^**6**^ (schools)	1,432	All children	7,671^**6**^ (schools)	1,432	All children	7,671^**6**^ (schools)	1,432
*Sub-total OHP including individualised prevention*					*43,940*			*42,711*			*54,911*
Restoration at tooth level	Atraumatic Restorative treatment (ART)	12.7	131,043	504,516	106,789	106,759	403,548	85,418	357,006	881,384	186,560
	Tooth filling	27.8	120,560	258,137	119,604	91,508	201,893	91,121	33,261	46,522	21,555
Surgical at tooth level	Tooth extractions	23.9 ^p^ /21.2^d^	29,703	52,277	20,824	18,640	24,419	9,727	86,479	171,274	60,637
Total clinical hours					291,157			228,977			323,663

^1,2^ Informed by Refs. [40]  + [42] + [43]  + Panel of members from the ICCMS, KCL and Public Sector Dentistry in SL

^3^ Ref. [41]

^4^ Total children in each of the three key age groups estimated from—Statistics Sierra Leone. 2015 Population and Housing Census. [Available from: https://www.statistics.sl/images/StatisticsSL/Documents/Census/2015/sl_2015_phc_thematic_report_on_pop_structure_and_pop_distribution.pdf. Accessed 15 Feb 2017]

^5^ Includes timings for treatment procedures for both permanent(^p^) and primary (^d^) teeth informed by 3

^6^ Total no. of schools informed by—Ministry of Education Science and Technology (MEST). Making Progress –Schools and Students in Sierra Leone. [Available from: http://wbgfiles.worldbank.org/documents/hdn/ed/saber/supporting_doc/AFR/Sierra%20Leone/ECD/SL%20SCR%202010_.pdf. Accessed on: 25 Sept 2018]

^7^ Two visits in a school in a year following—Public Health England (PHE). Delivering better oral health: an evidence-based toolkit for prevention. [Available from: https://assets.publishing.service.gov.uk/government/uploads/system/uploads/attachment_data/file/605266/Delivering_better_oral_health.pdf. Accessed 05 March 2020]. London: PHE 2017

**Table 4:**
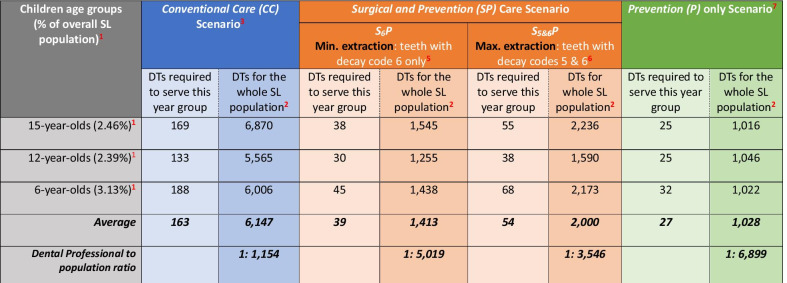
Estimated DTs required in 6-,12- and 15-year-olds and extrapolated for whole SL population according to different scenarios of care delivery

^1^Estimated population % of each age group of children within overall SL population (*n* = 7,092,113) informed by 2015 SL Census data

^2^Estimated dental therapists (DTs) calculated considering them working full time for 46 weeks in a year for overall SL population (*n* = 7,092,113)

^3^‘Conventional care’ scenario includes oral health promotion and prevention (shaded green), restoration (shaded orange) and extraction (shaded dark orange) as shown in Table 2

^4^‘Surgical and Prevention care’ scenario oral health promotion and prevention (shaded green) and extraction (shaded dark orange) only, as shown in Table 2

^5^Sub-type of ‘Surgical and Prevention’ scenario in which only teeth with decay code 6 will be managed with extraction along with oral health promotion and preventative strategy

^6^Sub-type of ‘Surgical and Prevention’ scenario in which teeth with decay codes 5 and 6 will be managed with extraction along with oral health promotion and preventative strategy

^7^‘Prevention care only’ scenario includes only oral health promotion and prevention (shaded green), as shown in Table 2, and no other treatment modality

When extrapolated to the whole national population, including adults, the model in the conventional (CC) scenario suggested the need for between 5565 and 6870 DTs nationally (Table 4). When maximum use was made of NDPs to deliver oral health promotion plus prevention (Table 5), the model suggested that the need for DTs was reduced to somewhere between 4,519 and 5,813 to deliver all conventional care.

**Table 5:** Estimated Non-dental personnel (NDP) and DTs required in 6-,12- and 15-year olds and extrapolated for whole SL population according to different scenarios of care delivery

Age groups	NDP^2^ delivering oral health promotion including individualised prevention	No. of DTs^3^ required across three scenarios of care			
		Conventional Care (CC) Scenario^4^	Surgical and Prevention (SP) Care Scenario		Prevention (P) only Scenario^6^
			*S*_*6*_*P* Min. extraction: teeth with decay code 6 only^5^	*S*_*5&6*_*P* Max. extraction: teeth with decay codes 5 and 6^5^	
15-year-olds (2.46%)^1^	127	143	12	29	NR*
12-year-olds (2.39%)^1^	124	108	6	14	NR*
6-year-olds (3.13%)^1^	159	156	13	36	NR*
Estimation for whole SL population extrapolated from 15-year olds data	5163	5813	488	1,179	NR*
Estimation for whole SL population extrapolated from 12-year olds data	5188	4519	251	586	NR*
Estimation for whole SL population extrapolated from 6-year olds data	5080	4984	415	1,150	NR*
Average estimation for the whole SL population	5144	5105	385	972//	NR*

^1^Estimated population % of each age group of children within overall SL population (*n* = 7,092,113) informed by 2015 SL Census data

^2^Non-dental personnel (NDP) including Community Health Officers (CHOs), Community Health Workers (CHWs), Traditional healers or School teachers estimated working part time (1 day a week) for 46 weeks delivering oral health care

^3^Dental therapists working full time (37.5 h per week) for 46 weeks

^4^DTs delivering ART, tooth fillings and extractions

^5^DTs delivering extractions only

^6^DTs not required (NR)* as NDPs delivering all oral health promotion including individualised prevention

### *Surgical and Preventive scenario (S*_*6*_*P and S*_*5&6*_*P)*

Within the basic *S*_*6*_*P* scenario, a total of 64,764 clinical hours for 15 year-olds, 52,438 for 12 year-olds and 78,128 for 6 year-olds were estimated to be needed for DTs to treat current oral disease in the respective age groups, as shown in Table 3. In the more extended *Surgical and Preventive care (S*_*5&6*_*P scenario)*, where all teeth with distinct and extensive decay into dentine were extracted, this amounted to higher total hours for each year-group: 94,232 h for 15 year-olds; 66,341 for 12 year-olds and 117,314 for 6 year-olds.

In the more conservative *S*_*6*_*P* scenario, the model estimated that 38 DTs would be required for 15 year-olds compared with 30 DTs to serve 12 year-olds nationally, whereas the workforce needs were highest for 6 year-olds at 45 DTs, as shown in Table 4.

A similar pattern was found for the *S*_*5&6*_*P* scenario, where the model estimated that 55 DTs would be required for 15 year-olds compared with 38 DTs to serve 12 year-olds nationally, and the workforce needs were again highest for 6 year-olds at 68 DTs, as shown in Table 4. This represented an average of 39 DTs per year-group for *S*_*6*_*P* scenario and 54 DTs per age cohort for the *S*_*5&6*_*P* scenario across the three age-groups investigated.

When extrapolated to the whole national population, as shown in Table 5, the *S*_*6*_*P* scenario suggested an average need of 1,413 DTs (range: 1255–1438 DTs) compared with 2,000 for the *S*_*5&6*_*P* scenario (range 1590–2236) (Table 4). However, if NDPs were to deliver oral health promotion and individualised preventive care, then the *S*_*6*_*P* scenario indicates an average of 385 DTs (range 251–488) for extracting teeth with extensive decay into dentine (D6) only. This rises to 972 DTs (586–1179) for extracting teeth with both distinct (D5) and extensive (D6) decay into dentine in the in the *S*_*5&6*_*P* scenario.

### Prevention only scenario (P)

The prevention only scenario for oral disease management, which included two visits to schools to deliver oral hygiene advice, diet advice, application of fluoride varnish and an oral examination for all schoolchildren, estimated a need of 43,940 clinical hours for 15 year-olds compared with 42,711 in 12 year-olds and 54,911 in 6 year-olds (Table 3).

The model estimated a requirement of 25 DTs to deliver preventive care in both 15 and 12 year-olds, whereas it was slightly higher for 6 year-olds with 32 DTs (Table 4). This represented an average of 27 DTs per year-group.

When extrapolated to serve the whole national population, the model suggested an average need of 1,028 DTs to deliver preventive care alone nationally (Table 4), or five times as many NDPs (*n* = 5144) if they contribute to oral health on 1 day per week (Table 5).

### Summary

In summary, when findings from the three key age groups are scaled up for the total SL population, the model estimated an average requirement of 6,147 DTs (range: 5565–6870) to deliver *Conventional care (CC)*; 1,413 DTs (range: 1255–1438 DTs) to deliver all necessary basic *Surgical and Preventive care (S*_*6*_*P)* in which teeth with extensive decay into dentine (D6) only are extracted, compared with 2,000 for the *S*_*5&6*_*P* scenario (range 1,590–2,236) in which teeth with decay codes D5 and D6 collectively are extracted; and 1,028 DTs to deliver *Prevention* only *(P)* (range: 1016–1046).

Furthermore, if task shifting is used to delegate oral health promotion, including individualised prevention to non-dental personnel, then the remaining surgical care could be delivered by 385 DTs (range: 251–488) for the *S*_*6*_*P* scenario which was deemed as the minimum basic service extracting all teeth with extensive caries into dentine and 972 DTs (range: 586–1179) for the *S*_*5&6*_*P* scenario in which all teeth with distinctive and extensive caries into dentine are extracted.

## Discussion

The findings from this study highlight the alarmingly high treatment and health promotion needs in Sierra Leone. Given the stark shortage of human resources for oral health to deliver basic dental care to tackle the most common oral disease, dental caries, the urgency of capacity building is evident. Our OR model demonstrates a requirement for around six thousand DTs to deliver *Conventional (CC)* care, of the nature that may be delivered in a high-income country; around two thousand DTs to deliver a mix of *Surgical and Preventive (S*_*5&6*_*P and S*_*6*_*P)* care; and around one thousand DTs to deliver *Prevention (P)* for the population. However, if non-dental personnel (NDPs) can contribute to deliver prevention, then the model suggested that this form of task shifting could reduce the need for trained dental professionals such as DTs, to an absolute minimum of around four hundred clinical personnel (*n* = 385 for the *S*_*6*_*P scenario*), rising to a more realistic provision approaching one thousand personnel for the *S*_*5&6*_*P scenario (n* = 972).

### Workforce needs, by age

The OR model also showed variation in total clinical hours estimated according to the type of treatment and year-group. The total clinical hours required to deliver all care together (Table 3) was highest in the 6 year-olds who have all their primary dentition and have begun the transition to a permanent dentition. Although the level of need appears to reduce in 12 year-olds, it rises steeply again in 15 year-olds. This pattern can be explained by the fact that 12 year-olds have just completed the transition from their deciduous to their permanent dentition. It does raise the question over whether disease in the deciduous dentition should be treated as these teeth will be lost [49, 50]; however, failure to provide clinical care will result in pain [51, 52], suffering [53, 54], days lost from school [55, 56], and in the case of SL even risk possible death [13] which is not acceptable for a preventable disease. Furthermore, by addressing the oral health needs of 6 year-olds holistically involving oral health promotion and prevention, they will be supported to have a better start in life and the risk of longer term oral disease may be reduced [57]. The clinical hours for delivering ‘oral health promotion and prevention’ were similar across year-groups. Such care is rather easier to deliver for school-age children but will be more challenging in relation to pre-school children and adults. None-the-less, SL has a young population [44], it may be possible to deliver this service to much of society using schools as community hubs, as advocated globally [58]. Whilst other options in most countries could include M-health involving mobile phones and delivering text messages [59, 60], the effectiveness of this approach is limited for caries prevention compared with fluoride varnish applications [61], or silver diamine fluoride [62], in addition to toothbrushing with a fluoride paste [63, 64], to lower the risk of dental caries.

### Validation of the model

To validate the estimates of DTs from this model and put the findings in a regional and global context, dental professional to population ratios were examined. The WHO data on the oral health workforce from the 46 African countries suggests that the ratio for all recorded dental professionals (dentists, DTs, dental hygienists, dental nurses, dental assistants, dental technicians collectively) is about 1:18,301 and the dentist to population ratio of and the dentist to population ratio is 1: 26,753 [65]. This ratio from the African region is considerably less than the global average of the dentist to population ratio of 1: 4,411 [66].

Among the three scenarios, the DT to SL population ratio in the *Conventional care (CC)* scenario was of the order of 1: 1,154 which was unsurprisingly similar to high-income regions of the world including Americas (1:1,440) and Europe (1:2,013) [66]. The *Surgical and Preventive scenario (SP)* demonstrated a DT to population ratio of 1: 3,546 in the more realistic scenario (*S*_*5&6*_*P)* and 1: 5,019 in the more basic scenario (*S*_*6*_*P)*, which are closer to the global average ratio [66]. Thus, overall, these levels of care suggested by the model may be considered realistic.

For SL, given that the current number of dentists (*n* = 10) and DCPs (*n* = 10) in SL (Table 1), the dental professional to population ratio stands at 1:375,000 which is critically low and highlights the prominent disparity when compared to either global or even the African region ratios. If NDPs are used to supplement the dental workforce, then the model suggested 385 DTs will be needed to deliver even the *S*_*6*_*P* scenario, to deliver a very basic or minimum baseline service in SL and translates into the dental professional to population ratio of 1: 18,421. This ratio is on a par with the African regional average of 1:18,301 [65], and could be a target for policymakers in SL as the first pragmatic goal in initiating capacity building for SL, working towards enhanced levels of care and equitable provision.

### The role of mid-level providers (DTs) and non-dental personnel (NDPs)

Evidence suggests that mid-level providers (such as DTs) can deliver significant primary care [67, 68], and could prove a more appropriate option especially in LICs [69], where there can be difficulties in developing, and retaining, highly skilled workers like doctors and dentists [70, 71]. The importance of using task-shifting and developing dental skill-mix to expand capacity in health care within Africa [72], and dental care in general, is increasingly recognized [68, 69], and has been undertaken in a number of countries including Cameroon [73], and Tanzania [74]. This would seem to be the most appropriate route in SL, and therefore, the OR modelling is based on DTs and, where possible, use of NDPs rather than dentists.

There is some evidence of the clinical effectiveness and cost-effectiveness of oral health promotion in dental caries prevention in children [75]. The role and effectiveness of school based oral health promotion and prevention programs in low resource settings is promising [76]. In our research, task-shifting of oral health promotion activities, including delegating individualised prevention, to NDPs including community health officers (CHOs), community health workers (CHWs), and schoolteachers was considered (Fig. 1). Hence, as NDPs are available in considerable numbers (Fig. 1), compared with qualified dental professionals in SL, and resembling the health workforce seen in most African countries, they are considered crucial in the WHO package for promoting oral health care [77]. The model estimated a total of just over 5,000 NDPs (range 5080–5188) will be required if working 1 day per week across a 46-week year for delivering OHP including individualised prevention.

Amongst the available options, the use of schoolteachers, would recognises that schools are natural hubs for care in society and present one possible route for innovation. However, their prime role is educational, and coverage may not always be adequate; sometimes in more remote areas there are challenges in retaining schoolteachers for educational purposes. Alternatives include community health workers and general health professionals; however, they too are very stretched, and their consideration of oral and dental issues is the exception rather than the rule. Whilst very supportive of a holistic approach, dental training is time intensive, and it is ultimately more cost-effective to train people who will stay in the discipline. There is also the potential for working with traditional healers if this is feasible; however, given that none has engaged with our dental research to date [13], further exploration will be required. Finally, community volunteers may be explored [78], although resources will be required to support their training and preventive care delivery.

In summary, using a mix of surgical and preventive care which could be delivered by DTs who can be trained more quickly, seems pragmatic at this stage to address the urgent needs within the country and reduce future disease. This should ideally go hand in hand with training NDPs and wider public health action [79, 80], as a part of the upstream measures and referral of complex care to trained dentists as part of whole systems integrated care [81]. These estimates assume that all trained personnel will be retained in SL. Challenges including their remuneration, motivation, retention and expectations must be taken into consideration [82–85].

### Vision for the future workforce

To achieve the above developments, a vision for a bespoke educational program for dental therapy in SL is being developed with a relevant scope of practice to facilitate access to care, within a wider oral health workforce plan.

First the vision has been that DTs will serve both children and adults, focusing on delivering surgical and preventive care, extending to simple restorative care when possible. Ideally one DT trainee should be selected from each of SL’s 16 districts per year to build a nationally representative workforce [39]. The findings from the OR model indicated an average requirement of between 27 and 163 DTs per year-group and somewhere between one and six thousand DTs for the population to manage current disease. It would, therefore, take more than 10 year-olds to train a workforce of DTs to address the basic restorative, surgical and preventive treatment needs of the three year-groups considered in this study and over 100 year-olds for all children and adults together, assuming a training period of two year-olds, treatments needs remaining constant, and 100% retention of the workforce. Although delivering conventional care which equates with a ‘westernised’ model might be aspirational for SL, it does not seem to be economically feasible or sustainable at present for a LIC [86].

**Fig. 3:**
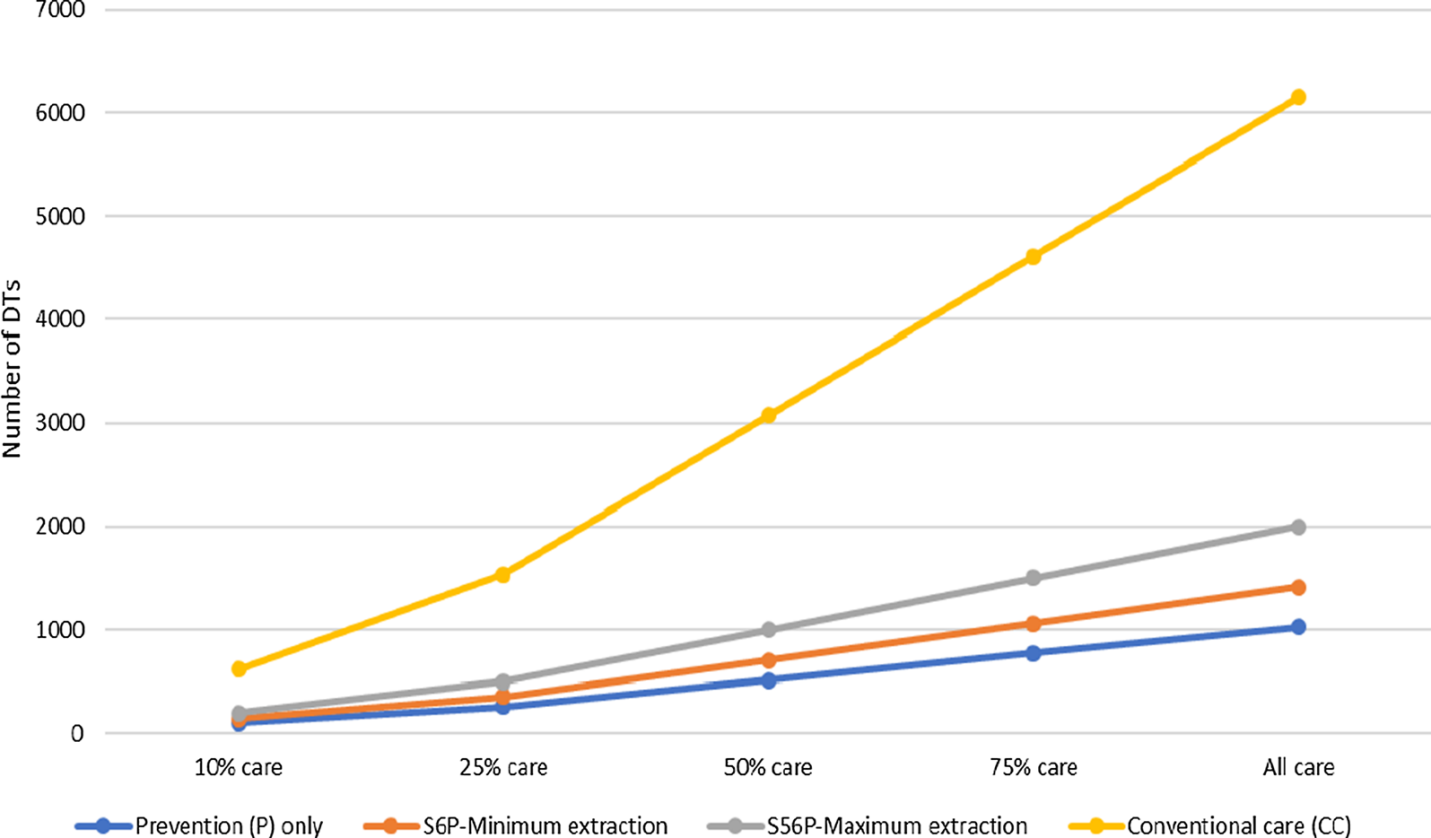
No. of DTs required to deliver different levels of care across Conventional (CC), Surgical and Prevention (*S*_*5&6*_*P and S*_*6*_*P*) and Prevention only (P) care scenarios

Second, it is well recognised that social disparity in accessing oral health care is evident in both high-and low–middle-income countries [87, 88], and it is, therefore, unrealistic not to expect the same in SL, particularly when there is a dental fee for care (young children and pregnant/nursing mothers exempted in the public sector). It is anticipated that as DTs are educated and trained it will be necessary to acculturate the population to the possibilities of care. It will take time to educate and training the workforce; thus, Fig. 3 which suggests the level of coverage by scenario type, two hundred DTs would be able to serve 10% of the population for the S_5&6_P scenario and if supported by NDPs they could provide clinical care for 20% in one year. Coverage will be supported by training people from each district of the country, and thus enabling them to undertake specific projects locally during their training, and employing them within their home district thereafter.

Third, the vision recognises that there is an important role for dentists in SL both in management of complex oral health needs and mentoring the mid-level providers including DTs. Serious oral conditions and diseases including osteomyelitis, Burkitt’s Lymphoma or Ludwig’s Angina are not uncommon in the country, and therefore, the role of dentists in delivering complex oral health care is vital. Furthermore, the views of dental professionals in SL indicated that there was a preference for urgent (pain-relieving treatment mainly of tooth extraction) oral care [13]. Further thought needs to be given to how best to ensure a small number of dentists, including specialists who can prioritise more complex care, may be educated and trained for the national workforce, possibly with the support of local nations.

Fourth, the scale of dental disease reported in SL [45], emphasises the use of a population or dental public health approach to caries management. The availability and increased intake of sugars in SL [13], given dietary trends in Africa towards a more ‘westernised’ diet involving free sugars and processed foods [89–91]. Although undernutrition and hunger still persist in Africa, the increase in the rate of non-communicable diseases (NCDs) in the region is documented [7]. It is evident, and acknowledged, that oral diseases and inequalities in oral health are based on a variety of complex interplay of factors including individual, social, economic, environmental, and political determinants which are also related to other NCDs [81, 92]. Therefore, it warrants strong public health leadership to ensure action on upstream measures [79, 80, 93, 94].

### Strengths and limitations

A recent study by Ahern et al. [28], highlighted the fact that most countries that engage in model-based workforce planning do not take into account the oral health needs of the population and there is a scarcity of data available to operationalise models specifically for oral health care. The current study, therefore, makes a major contribution to the literature in considering in detail the oral health needs of the SL population to estimate a baseline requirement of providers. The bespoke OR model developed for this study focused on management of dental caries, the most common oral condition [13, 45]. However, there are several limitations to this study which need to be acknowledged.

First, oral health surveys generally underestimate need and there is a further possibility that an underestimation of clinical hours might have occurred using this OR model which was largely based on caries management and not considering periodontal disease and the wider range of serious conditions affecting the population [13]. However, in relation to the most common of these conditions, periodontal disease, the oral health promotion and preventative measures included in the model involved an emphasis on toothbrushing with a fluoride toothpaste. Thus, having considered these issues, additional time was not factored into the model as it was felt that this would be sufficient to address basic periodontal health needs [45]. Whilst this could be argued as sufficient for children, it may not be for adults; thus overall, this model is likely to underestimate needs. None-the-less, an adult dental health survey will be required for more appropriate estimates of oral health workforce.

Second, clinical timings are based on UK research and practice of both DTs and dentists [30, 41], and the scope of practice of DTs has been expanded to include competence in removal of permanent teeth on which there are no available data. Given the clinical context in SL [13], they could be slower than their UK counterparts or find innovative ways of delivering care quickly. Validation is not possible unless information on clinical timings of DTs with this scope of practice working in SL or other comparable LICs are available.

Third, population data used in the model were based on the 2015 census [44], which was the most recent evidence available. The workforce estimates for the wider SL population are extrapolated from the three key age groups and thus, additional data on adults will be required for more accurate projections. Having surveyed the three key ages of children recommended by the WHO [95], these data do provide insight to the changing needs over time and suggest that the rise in oral health needs into adulthood may be significant unless urgent action is taken.

Fourth, the model assumes all clinical care will be treated; however, evidence from SL highlighted that uptake of care is low, mainly focused on urgent pain-relieving treatment, and mostly available in the capital city. In addition, it does not consider health workforce turnover and retention [96–98], all of which need to be taken account of in a more advanced model [35]. Given that it will take time to train a dental workforce and explore the potential for training and using NDPs, this will provide the opportunity to simulate uptake and promote dental attendance and adjust workforce numbers accordingly.

Fifth, it will also be important to investigate the oral health needs of adults and determine how realistic the projections of healthcare and workforce needs of children can be extrapolated to the population and test the requirements for capacity building through mid-level and non-dental personnel in SL. Similarly, it will be essential to explore the uptake of care as dentistry becomes available. Finally, it should be possible to apply this model to other low- and middle-income countries.

Despite its limitations we argue that this simple model is helpful to work with planners and policy makers in a country, where there is almost a blank canvas in relation to developing an oral and dental workforce. Furthermore, it is a model that could be used across all LICs, where survey data are available. There is growing support amongst policy makers to address the pressing oral health needs and build capacity. The importance of co-production initiatives to improve health and wellbeing is fundamental to addressing health inequity [99].

### Moving to action

We have worked collaboratively to produce a bespoke costed training curriculum for DTs and linked with a range of agencies to explore how best to take this forward. The global pandemic has currently halted further developments but is providing the opportunity to plan for a better future. This project will require significant financial investment, together with academic support. None-the-less, given the potential for the generation of fees through public sector services; it should, in time, become at least partially self-sustainable. The process will require supportive policies including publication of a transparent ‘scope of practice’ for DTs and any NDPs involved in the delivery of care. Whilst there have been several DTs in the public sector, it will be particularly important that this branch of the workforce is well integrated in the staffing and remuneration systems for the public sector, with an appropriate role and grade, to ensure that they are easily incorporated into the workforce once trained.

Extended task shifting will be evaluated to determine the potential for innovation using NDPs. Realistically, whilst we would ideally love others to be advocates for oral health, we may find that teachers with important educational requirements and community health workers multiple pressures will not be able to support oral health promotion initiatives or traditional healers may resist engagement; thus, we will have to test in a stepwise approach what other players can contribute including community volunteers. It may be that in different parts of the country alternative solutions will emerge.

## Conclusion

Our research highlights the dental workforce needs required to deliver even minimal oral health care to the Sierra Leone population. The gap between the current workforce and the oral health needs of the population is stark and requires urgent action. For oral disease management using mid-level providers with a locally appropriate skill base, the model suggests an estimated requirement of around two thousand mid-level dental providers to deliver surgical and preventive care for the whole country in the first instance. However, if non-dental personnel (NDPs) are trained to deliver oral health promotion and prevention, the estimated number of DTs needed was reduced. The study also demonstrates the potential for contemporary epidemiological tools to predict dental treatment needs and inform workforce capacity building in a low-income country, exploring a range of solutions involving mid-level providers and non-dental personnel.

## Data Availability

The data sets generated during and/or analysed during the current study are not publicly available at this time.

## Abbreviations

ART: Atraumatic restorative treatment
CHOs: Community health officers
CHWs: Community health workers
COMAHS: College of Medicine and Allied Health Sciences
DCPs: Dental care professionals
DTs: Dental therapists
EVD: Ebola virus disease
FTE: Full-time equivalent
HRH: Human resources for health
ICDAS: International Caries Detection and Assessment System
ICCMS: International Caries Classification and Management System
KCGH: King’s Centre for Global Health
LICs: Low-income countries
NDP: Non-dental personnel
OR: Operational research
PUFA: Pulp, Ulceration, Fistula, Abscess
SL: Sierra Leone
UK: United Kingdom
WHO: World Health Organization
